# Hemobilia following Percutaneous Liver Biopsy in the Setting of Malignancy (with Video): Diagnosis and Management of a Rare Cause of Upper Gastrointestinal Bleeding

**DOI:** 10.1155/2019/3087541

**Published:** 2019-02-17

**Authors:** B. Pabla, M. Porayko

**Affiliations:** Division of Gastroenterology, Hepatology, and Nutrition, Vanderbilt University Medical Center, USA

## Abstract

A 57-year-old gentleman with a past medical history of well-differentiated pancreatic neuroendocrine tumor (NET) with liver metastases was transferred to our hospital with abdominal pain. He underwent percutaneous liver biopsy three days prior to admission as a part of a study protocol for treatment of his progressive NET. He developed gastrointestinal bleeding and was found to have a distended gallbladder filled with high density material on ultrasound. During initial upper endoscopy, it was noted that he had blood emanating from the duodenal papilla consistent with hemobilia and he was ultimately diagnosed with post-liver biopsy hemorrhage. At first, he was managed conservatively with supportive care, but bleeding persisted resulting in the need for arterial embolization as a more effective treatment modality. Hemobilia is a rare entity and in the modern era it is most commonly the result of iatrogenic injury. Appropriate management depends on the underlying etiology with most cases resolving with conservative management. The avoidance of unnecessary surgery and the use of embolization are key principles in management.

## 1. Case Presentation

A 57-year-old gentleman with a past medical history of well-differentiated pancreatic neuroendocrine tumor (NET) with liver metastases was transferred to our hospital with abdominal pain. He presented to an outside hospital three days after undergoing a diagnostic ultrasound-guided percutaneous liver biopsy as part of his participation in an experimental treatment protocol. Following the biopsy, he developed worsening of a chronic right upper quadrant abdominal pain, now coming in waves and radiating throughout the abdomen. He denied having any fevers, nausea, vomiting, or signs of gastrointestinal bleeding. However, he had not had any bowel movements for several days prior to presentation even though he continued to pass flatus. He had been diagnosed with a well-differentiated neuroendocrine tumor about seven years prior to this admission and had several hepatic metastases, making him a nonoperative candidate. He failed multiple treatment modalities including octreotide, Afinitor, pazopanib, Temodar, capecitabine, and temozolomide in combination with Y-90 and bland embolization.

**Figure 1:**
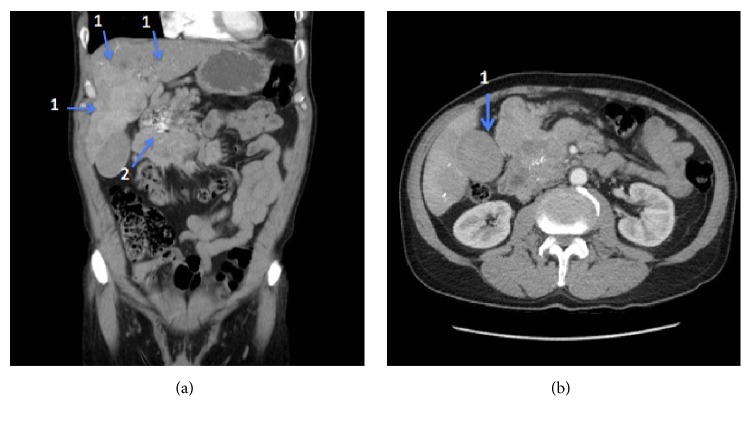


Upon presentation, his vitals were unremarkable. Pertinent labs revealed liver enzyme elevations including an AST 524 U/L, ALT 614 U/L, alkaline phosphatase 224 U/L, and total bilirubin 5.0 mg/dL. Other labs were notable for a normal white blood cell count and lipase, as well as hemoglobin 13.6 gm/dL. A computed tomography scan of the abdomen and pelvis revealed diffuse hepatic metastatic disease (Figure 1(a), arrow 1) with a slight increase in disease burden when compared to a scan obtained one week previously. Additionally, a lobulated partially calcified pancreatic mass (Figure 1(a), arrow 2) with sequela of prior embolization was unchanged and the gallbladder was distended with high density material in the lumen without gallbladder wall thickening, pericholecystic stranding, or pericholecystic fluid (Figure 1(b), arrow 1). There was no evidence of bile duct dilatation. His hemoglobin decreased to 10.2 gm/dL without any signs of gastrointestinal bleeding and his liver enzymes remained abnormal; therefore he was transferred to our institution for further management. His hemoglobin continued to decrease, but he started having melena, indicating the source of blood loss. Upper endoscopy revealed blood clots emanating from the papilla with fresh blood in the duodenum (Video 1).

This patient was diagnosed with gastrointestinal blood loss after liver biopsy without evidence of a vascular fistula. Initially, he was managed conservatively with consultative help from hepatobiliary surgery and interventional radiology. He was discharged after his hemoglobin stabilized. However, he was readmitted with recurrent melena and underwent upper endoscopy with EUS but neither extravasation of blood from the biliary tree nor a vascular fistula was found. Repeat CT angiography of the abdomen was negative for active bleeding. An empiric embolization of retroduodenal/retroportal collaterals arising from the stump of the GDA (the patient had previously undergone GDA embolization for his NET approximately 1 year prior to presentation) but he continued to bleed. Repeat embolization of the right hepatic artery was performed with focused treatment of a segment V arterial branch, presumed to be supplying the territory of the previous liver biopsy. The patient's bleeding resolved and his liver enzymes stabilized. He was discharged from hospital and remained in good condition on follow-up in the outpatient clinics.

## 2. Discussion

Gastrointestinal hemorrhage following liver biopsy was first reported in 1967, but only a few cases have been reported in the literature since that time [1–5]. Patients typically present with one or more symptoms of Quincke's triad, jaundice, upper abdominal pain, and obscure GI bleeding. Contemporary trials of percutaneous liver biopsies with ultrasound guidance list bleeding as the most common adverse event at a rate of 0.6%, with hemobilia occurring in 0.1% of patients [6]. Hemobilia following liver biopsy can be either arterial or venous in origin and in rare instances can result in the formation of an arterioportal fistula with the development of portal hypertension [7]. In cases where there is concern for the development of new onset portal hypertension, multiphasic computed tomography or EUS can be performed to assess the presence of an arterioportal fistula. Though older retrospective studies have noted an association between malignancy and post-liver biopsy hemorrhage, more contemporary studies have failed to confirm this association [8, 9]. In this case, the risk of bleeding following liver biopsy may have been elevated as primary pancreatic neuroendocrine tumors and their metastases are often hypervascular [10]. The decision regarding the need for vascular surgical or radiological intervention should continue to be guided by clinical course. Case series have been written describing successful management of persistent bleeding with vascular embolization [11]. While hemobilia can lead to biliary obstruction with resultant cholangitis or cholecystitis which may require ERCP for management, this appears to be a rare consequence of accumulated blood in the biliary tract [12]. More recent experience has demonstrated that conservative management without cholecystectomy is adequate in most patients, as blood in the gall bladder and biliary system is often evacuated spontaneously over time [9, 13]. Attempts to stop bleeding by endoscopic biliary stent placement are not likely to be helpful for hemorrhage after liver biopsy.

## Supplementary Materials

Supplementary Materials Video obtained during upper endoscopy with standard gastroscope demonstrating blood clots and fresh blood being extruded from the major papilla.
